# Quantum chemical calculation studies toward microscopic understanding of retention mechanism of Cs radioisotopes and other alkali metals in lichens

**DOI:** 10.1038/s41598-021-87617-w

**Published:** 2021-04-15

**Authors:** Hiroya Suno, Masahiko Machida, Terumi Dohi, Yoshihito Ohmura

**Affiliations:** 1 Center for Computational Science & e-Systems (CCSE), Japan Atomic Energy Agency, 178-4-4 Wakashiba, Kashiwa, 277-0871 Japan; 2 Sector of Fukushima Research and Development, Japan Atomic Energy Agency, 10-2 Fukasaku, Miharu-machi, Tamura-gun, Fukushima 963-7700 Japan; 3 Department of Botany, National Museum of Nature and Science, 4-1-1 Amakubo, Tsukuba, Ibaraki 305-0005 Japan

**Keywords:** Biochemistry, Molecular biology, Physiology, Environmental sciences, Chemistry, Mathematics and computing

## Abstract

We evaluate stability of cesium (Cs) and other alkali-metal cation complexes of lichen metabolites in both gas and aqueous phases to discuss why lichens can retain radioactive Cs in the thalli over several years. We focus on oxalic acid, (+)-usnic acid, atranorin, lecanoric acid, and protocetraric acid, which are common metabolite substances in various lichens including, e.g., *Flavoparmelia caperata* and *Parmotrema tinctorum* retaining Cs in Fukushima, Japan. By performing quantum chemical calculations, their gas-phase complexation energies and aqueous-solution complexation free energies with alkali-metal cations are computed for their neutral and deprotonated cases. Consequently, all the molecules are found to energetically favor cation complexations and the preference order is Li$^+>$Na$^+>$K$^+>$Rb$^+>$Cs$^+$ for all conditions, indicating no specific Cs selectivity but strong binding with all alkali cations. Comparing complexation stabilities among these metabolites, lecanoric and protocetraric acids seen in medullary layer are found to keep higher affinity in their neutral case, while (+)-usnic acid and atranorin in upper cortex exhibit rather strong affinity only in deprotonated cases through forming stable six atoms’ ring containing alkali cation chelated by two oxygens. These results suggest that the medullary layer can catch all alkali cations in a wide pH range around the physiological one, while the upper cortex can effectively block penetration of metal ions when the metal stress grows. Such insights highlight a physiological role of metabolites like blocking of metal-cation migrations into intracellular tissues, and explain long-term retention of alkali cations including Cs in lichens containing enough such metabolites to bind them.

## Introduction

Since lichens have no root apparatus, their mineral nutrition depends mainly on atmospheric inputs. In addition, owing to the lack of protective cuticle and stomata, thalli accumulate mineral elements including heavy metals at levels far exceeding their metabolic requirements^1–3^. Consequently, they can grow even in heavily contaminated areas or highly-toxic geographical ones showing high concentrations of uranium, copper, lead, arsenic phosphates, and so on. Lichens have been also known to retain radionuclides including radioisotopes of Cs. Indeed, high-level of $^{137}$Cs radioactivity was detected in lichens collected during and after nuclear weapons test period^4–6^ as well as after nuclear reactor accidents in Chernobyl^7–11^ and Fukushima^12–15^. In addition, they have been known to be able to keep $^{137}$Cs for much longer time than the other biological species^4,8,16^.

Cell walls of lichen-forming fungi are supposed to initially catch $^{137}$Cs at nuclear events. Subsequently, $^{137}$Cs is restored in extracellular space by forming complexes with secondary metabolites of the fungal partner^17^, which are distributed on the surface of the hyphae rather than inside the cells. Thus, lichens have been widely used as biomonitors of $^{137}$Cs and the other radionuclides as well as toxic heavy metals.

As lichens grow under high concentration conditions of toxic metals on such as rocks and minerals^18^, the formation of metal oxalates and lichen acid-metal complexes has been regarded to help lichens to tolerate high concentrations of toxic metals when growing on certain rocks and minerals. Indeed, Sarret et al.^19^ argued the idea through the experimental investigations on Pb and Zn with lichen metabolites, and afterwards, a number of literatures^1,3,18–21^ supported it. For other polyvalent metals such as Mg, K, Fe and so on, lichen metabolites are also known to possess functions as metal chelators by forming complex with their ions^21^. Moreover, lichens have been known to have species dependence on metal accumulation ability. Generally, the ability mainly depends on their metabolite production performance, which is a kind of classification indices of species, while the amount of some substances would vary depending on their living environments. Despite these several intriguing findings, the details of metal complexation of lichen metabolites have remained elusive. In this paper, we therefore focus on the complexation mechanisms of lichen metabolites with Cs and other alkali cations.

Aside from biochemical roles described above, lichen metabolites have been confirmed to be also widely useful in pharmaceutical applications^22–28^. For example, some metabolites are now known to possess several useful properties like UV-light shielding, antibiotic, anti-proliferative, anti-inflammatory, and anti-oxidant ones^29^. Some of these properties can be attributed to their molecular structures like polyaromatic structures containing multiple hydroxyl groups. The presence of multiple hydroxyl groups is also considered to significantly promote the metal complexations of their metabolites.

**Figure 1:**
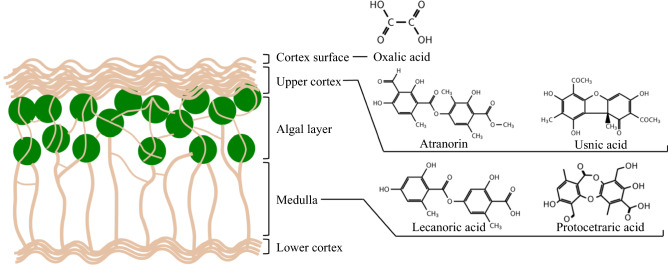
Schematic internal structure of some lichen species with distribution of the lichen metabolites considered in the present work: oxalic acid, atranorin, lecanoric acid, usnic acid, and protocetraric acid.

So far, more than 1050 lichen substances have been known^30^, and most of them have been found to be unique to lichens, with only a small minority 50–60 occurring in other fungi or higher plants^31^. In this work, we choose five lichen substances, i.e., oxalic acid (C$_2$H$_2$O$_4$), atranorin (C$_{19}$H$_{18}$O$_8$), lecanoric acid (C$_{16}$H$_{14}$O$_7$), (+)-usnic acid (C$_{18}$H$_{16}$O$_7$) (we note simply usnic acid hereafter), and protocetraric acid (C$_{18}$H$_{14}$O$_9$) as shown in Fig. 1. These are found commonly in parmelioid lichen species seen in Fukushima^15^, e.g., *Flavoparmelia caperata* (L.) Hale and *Parmotrema tinctorum* (Nyl.) Hale, which we will note respectively *F. caperata* and *P. tinctorum* below. Among them oxalic acid is a primary metabolite while others are secondary metabolites. These substances are common and produced not only in parmelioid lichens but also in many other lichens^32–34^.

Figure 1 shows a typical internal structure of some lichen species, which consist of lower cortex, medulla, algal layer, upper cortex, and surface from the bottom to the top, together with distribution of the lichen metabolites examined in the present work. Generally, combinations of secondary compounds are species-specific. Then, the fact has been widely used in lichen taxonomy and systematics^35^. For example, *F. caperata* contains usnic acid in the upper cortex and protocetraric and caperatic acids in the medulla^36^, and *P. tinctorum* contains atranorin in the upper cortex and lecanoric acid in the medulla^37^. Substances in the upper cortex have a primary biological role as light-screen pigments, regulating the solar irradiation reaching to the internal layers^31^, while they have various other useful properties, one of which is metal complexation ability. In contrast, lots of the colorless depsides and depsidones are produced in the medullary layer, including lecanoric acid (depside) and protocetraric acid (depsidone). In *P. tinctorum*, amounts of lecanoric acid in the range 24–37% dry weight were detected^38^. Lecanoric, protocetraric, and usnic acids have been characterized for antibiotic ability^39,40^ and metal homeostasis^41,42^, while other different roles are not known well, yet. Then, we study the alkali-metal-cation complexes of five lichen metabolites for their neutral and deprotonated molecules to cover their complexation stabilities in weakly acidic to alkaline conditions around physiological pH. Here, we note that Fig. 1 just schematically displays structures of some lichen types, e.g., there are species having no lower cortex. In addition, distributions of these metabolites become not so clear but somewhat vague in different species.

In this paper, we perform quantum chemical calculations in order to obtain the stable molecular structures and compute the energies and free energies for these alkaline metal complexes both in gas and aqueous phases, respectively. Here, we stress that since lichens are known to absorb alkali metal cations even in contact with the gas phase being one of unique characters of lichens, metal complexation stabilities in the gas phase should be informative. Moreover, we point out that these metabolite molecules exist abundantly as alkali and other metal salts in the intercellular spaces. Among these salts, only usnic acid salts were particularly examined in details in atomic level motivated by its pharmaceutical usefulness. However, such studies are still insufficient to conclude the complex structures, since all of them were not supported by theoretical calculations taking into account varieties of complexation due to multiple hydroxyl groups in quantum chemical levels^22–27^. Thus, we perform quantum chemical calculations on their deprotonated molecules as well as neutral ones. The calculations in the deprotonated condition are directly relevant with their salt formation stabilities.

Our quantum-chemical computational technique is a stepwise approach developed recently by Ota et al.^43^: the first step combines the semi-empirical PM6 (parametric model 6) method^44^ and the multicomponent artificial force induced reaction (MC-AFIR) method^45,46^ to screen stable reaction-product structures among several candidates, the second step subsequently uses the density functional theory (DFT) to optimize the screened candidate structures in ab initio quantum mechanical level, and the third and final step combines DFT with the polarizable continuum model (PCM)^47,48^ to take into account the solvation effects. This approach was successfully applied to study the alkali-metal-cation complexation selectivity of norbadione A, an important pigment in mushrooms^49^.

## Methods and computational details

**Figure 2:**
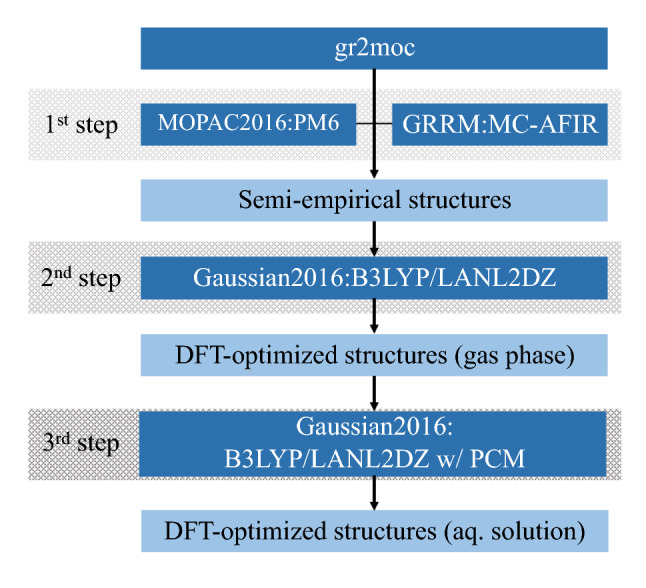
Illustration of the computational procedure.

Our computational procedure is illustrated in Fig. 2. Since the employed computational procedure was detailed in Ref.^43^, we give here only a brief outline. We perform so-called screening tests using PM6 implemented in the MOPAC 2016 program^44^ together with MC-AFIR implemented in the GRRM 14 program^50^. Here, we utilize an interface program between GRRM 14 and MOPAC 2016, gr2moc developed by Ota et al.^43^. MC-AFIR finds different associative pathways from randomly generated configurations of reactants by applying multiple artificial forces between intra- and inter-reactant components. The structure of the free (uncomplexed) molecule is prepared by optimization at DFT B3LYP^51,52^/6-31G(d,p)^51–64^ level using Gaussian 16^65^. In practice, an artificial force is applied between the cation and each oxygen atom of the molecule, since the oxygen atoms possess high electronegativity inside the molecules. The upper bound of the model collision energy between the cation and the oxygen atom used in MC-AFIR is set to be 100 kJ/mol.

The structures discovered in this screening procedure are then independently reoptimized at DFT B3LYP/LanL2DZ^66–69^ level using Gaussian 16. The structures in the solution phase are obtained by optimization further at DFT B3LYP/LanL2DZ level with PCM, starting from the above gas-phase optimized structures. We can study the statistical properties in solution at a temperature *T* by using the canonical ensemble of the stable structures. Then, the partition function is expressed as

1 $$\begin{aligned} Z_{\mathrm {PCM}}=\sum _{s}e^{-E^{(s)}_{\mathrm {PCM}}/k_{\mathrm {B}T}}, \end{aligned}$$

with $k_{\mathrm {B}}$ being the Boltzmann constant, and the summation index *s*, identification number in ascending order with increasing energy, runs over the distinct equilibrium structures. The free energy can be evaluated from the partition function as $A_{\mathrm {PCM}}=-k_{\mathrm {B}}T\ln Z_{\mathrm {PCM}}$, allowing us to take into account the emergence of different structures with energetically proximate to the most stable state in solution phases. Throughout this work, these all quantities are calculated at room temperature $T=300$K. Thus, the stability of a molecule-cation complex LM$^+$ in the gas and in aqueous solution phases is quantified, respectively, by the complexation energy difference:

2 $$\begin{aligned} \Delta E^{\mathrm {cmplx}}_{\mathrm {gas}} = E({\mathrm {LM}}^+)-E({\mathrm {L}})-E({\mathrm {M}}^+), \end{aligned}$$

and the free energy difference

3 $$\begin{aligned} \Delta G^{\mathrm {cmplx}}_{\mathrm {aq}} = A_{\mathrm {PCM}}({\mathrm {LM}}^+)-A_{\mathrm {PCM}}({\mathrm {L}})-A_{\mathrm {PCM}}({\mathrm {M}}^+). \end{aligned}$$

From these definitions, we can evaluate that the more negative the energy or free energy is, the more strong or favored the complexation is. The accuracy of our calculations is checked by estimating the counterpoise corrections. For all the complexes considered here, the corrections are found to be less than 1 kcal/mol, so that the BSSEs (basis set superposition errors) can be considered to be negligibly small compared with the energies calculated below.

The advantage of our approach over other quantum chemical methods is that we can obtain, in an unbiased and automated way, a number of stable and metastable structures associated with different minima on the potential energy surface. This allows us to calculate the free energies and to predict experimentally observable quantities on cation complexation, such as the cation-complexation free energies in aqueous phases. In lichen metabolites, using GRRM is significantly advantageous, because tautomerism frequently plays an important role for their biological activities in aqueous phases. The tautomers are well-known to mainly occur by intra-molecular hydrogen migration in these metabolites. Especially, usnic acid is a famous molecule showing keto-enol tautomerism and the interconversion easily occurs depending on the solvent^70–72^. Our approach can fully cover such tautomerism with their tautomer’s complexation stabilities in automatic manner. GRRM method allows to find the ground and higher stable complexes with its tautomers and take them into consideration in free energy calculations. For tautomerism, see the Supplemental Information Section 1 for usnic acid as a typical example. In addition, we would like to stress that, due to the combination of GRRM with MOPAC, we can obtain, with quite low computational costs, initial guesses of multiple energetically-ordered structures from the lowest to subsequent ones. We can also perform the subsequent DFT reoptimization with sufficiently low costs, because good initial states are already given in the first screening step.

## Calculation results

Since the alkali-metal-cation complexation mechanism are different between the neutral and the deprotonated molecules as we see below, we shall divide this section into the following two different subsections: neutral molecule complexation and deprotonated molecule complexation. In general, the neutral molecule dominates in pH $<{\mathrm{pK}}_{a} \, (<{\mathrm{pK}}_{a1}$ in polyprotic acid) for target molecules, while the deprotonation occurs in pH$\gtrsim$pK$_{a1}$. pK$_a$’s of some typical metabolites are known in the literature^28,73–76^. Thus, we discuss complexation stabilities of the metabolites mainly in physiological pH range around pH$\sim$7.4 ± 1.5 with literature information of pK$_a$’s of the present target molecules seen in Fig. S2. Since di-deprotonated states also occur in physiological pH range for two molecules (oxalic acid and atranorin) as seen in Fig. S2, calculation results for complexes of their di-deprotaned states with alkali cations are presented in Figs. S3 and S4.

### Neutral molecule complexation

**Figure 3:**
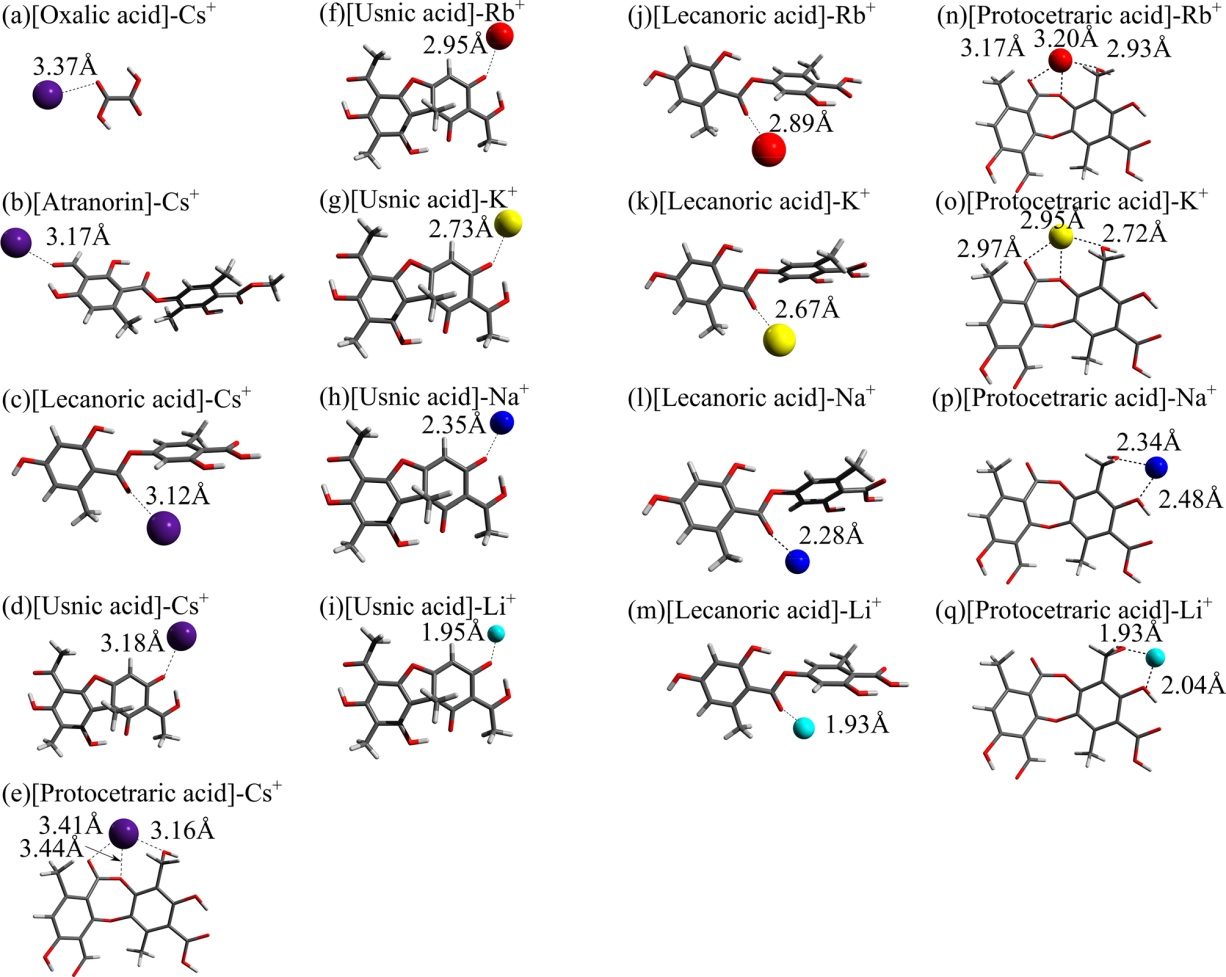
The most stable structures for the Cs-cation complexes of the lichen neutral metabolites: (**a**) oxalic acid, (**b**) atranorin, (**c**) lecanoric acid, (**d**) usnic acid, (**e**) protocetraric acid as well as those for the usnic acid complexed with (**f**) Rb$^+$, (**g**) K$^+$, (**h**) Na$^+$, and (**i**) Li$^+$, the lecanoric acid complexed with (**j**) Rb$^+$, (**k**) K$^+$, (**l**) Na$^+$, and (**m**) Li$^+$ and the protocetraric acid complexed with (**n**) Rb$^+$, (**o**) K$^+$, (**p**) Na$^+$, and (**q**) Li$^+$ in aqueous phase. The distances from the alkali-metal cation to the nearest oxygen atoms of the molecule are also indicated.

The most stable structures for the Cs-cation complexes of the lichen-metabolite neutral molecules in aqueous phase are displayed in Fig. 3a–e. The distances between the Cs cation and the nearest oxygen atom coordinated to it are also given. For the molecules considered in this work except protocetraric acid, Cs$^+$ cation is initially coordinated to a single oxygen atom. This allows chelating formations through approach of adjacent oxygen atoms by optimization processes. Generally, the Cs$^+$-O distances are found to be between 3.1 and 3.5 Å as shown in Fig. 3a–e. For usnic acid, lecanoric acid, and protocetraric acid, Fig. 3f–i,h–m,n–q show their complexed structures with Rb$^+$-Li$^+$ cations, respectively. Except protocetraric acid, all the alkali-metal cations are found to be coordinated with the same single oxygen atom irrespective of cation-size difference. The cation-oxygen distances are shorter for lighter alkali-metal cations. We find that these findings also hold for the other two molecules, oxalic acid and atranorin. For protocetraric acid, Cs$^+$, Rb$^+$, and K$^+$ are seen to be coordinated with three oxygen atoms, while Na$^+$ and Li$^+$ are chelated by two oxygen atoms. This peculiarity of protocetraric acid stems from its dense oxygen configuration inside the molecule.

**Figure 4:**
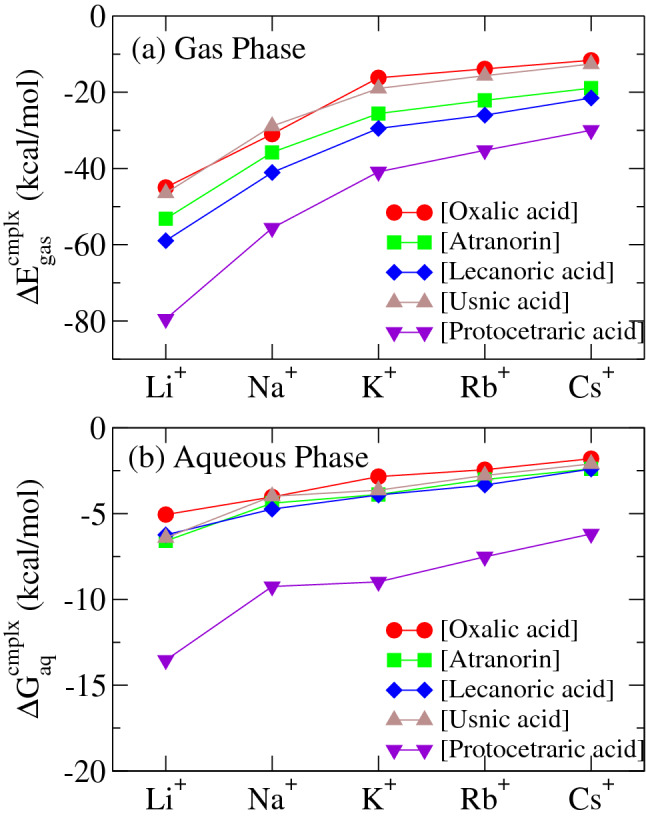
(**a**) Gas-phase complexation energies of the oxalic acid, atranorin, lecanoric acid, usnic acid , and protocetraric acid molecules with the alkali-metal cations. (**b**) Aqueous-solution complexation free energies between the same molecules and the alkali-metal cations. The above both quantities are computed for neutral metabolite molecules with alkali cations.

The gas-phase complexation energies $\Delta E^{\mathrm {cmplx}}_{\mathrm {gas}}$ and the aqueous-solution complexation free energies $\Delta G^{\mathrm {cmplx}}_{\mathrm {aq}}$ for all the metabolite—alkali-metal-cation complexes are shown in Fig. 4a and b, respectively. All the gas-phase complexation energies are found to become more negative and larger for lighter alkali-metal cations, indicating that all the metabolite molecules favor complexation with lighter cations. Such a trend is also kept for the aqueous-solution complexation free energies. All the molecules also favor complexation with lighter cations in aqueous phase. For a given alkali-metal cation, differences of the complexation free energies among the metabolites are also nearly preserved in hydrated conditions.

Comparing the complex formation ability among metabolites, we find that, only for protocetraric acid, the alkali-metal is coordinated by multiple oxygens, while the coordination number is 1 for the other four lichen metabolite species in both phases. This implies that protocetraric acid would be much more effective for complexations with alkali cations in its neutral structure. However, the neutral one of protocetraric acid is dominant in low pH range as seen in Fig. S2. In addition, it is found that the metal complexation stability of lecanoric acid are slightly larger than the remaining three metabolites except for protocetraric acid. We can see in Fig. S2 that the dominant pH range of the neutral structure of lecanoric acid fully covers the physiological one. These results suggest that medullary metabolites (lecanoric acid and protocetraric acid) can effectively catch alkali metal ions in their neutral molecular forms, which occur widely from low to physiological pH.

### Deprotonated molecule complexation

In the previous subsection, we considered the alkali-metal-cation complexes of five lichen metabolites in their neutral forms. Here, we point out that these molecules inside lichen bodies are known to exist abundantly as the form of alkali-metal salts, in which one proton belonging to an –OH group of the molecule is replaced by an alkali-metal cation, like potassium usnate for usnic acid^77^. Thus, the alkali-metal salts are regarded to be formed through the complexations of the mono-deprotonated lichen metabolites with the corresponding alkali-metal cation.

**Figure 5:**
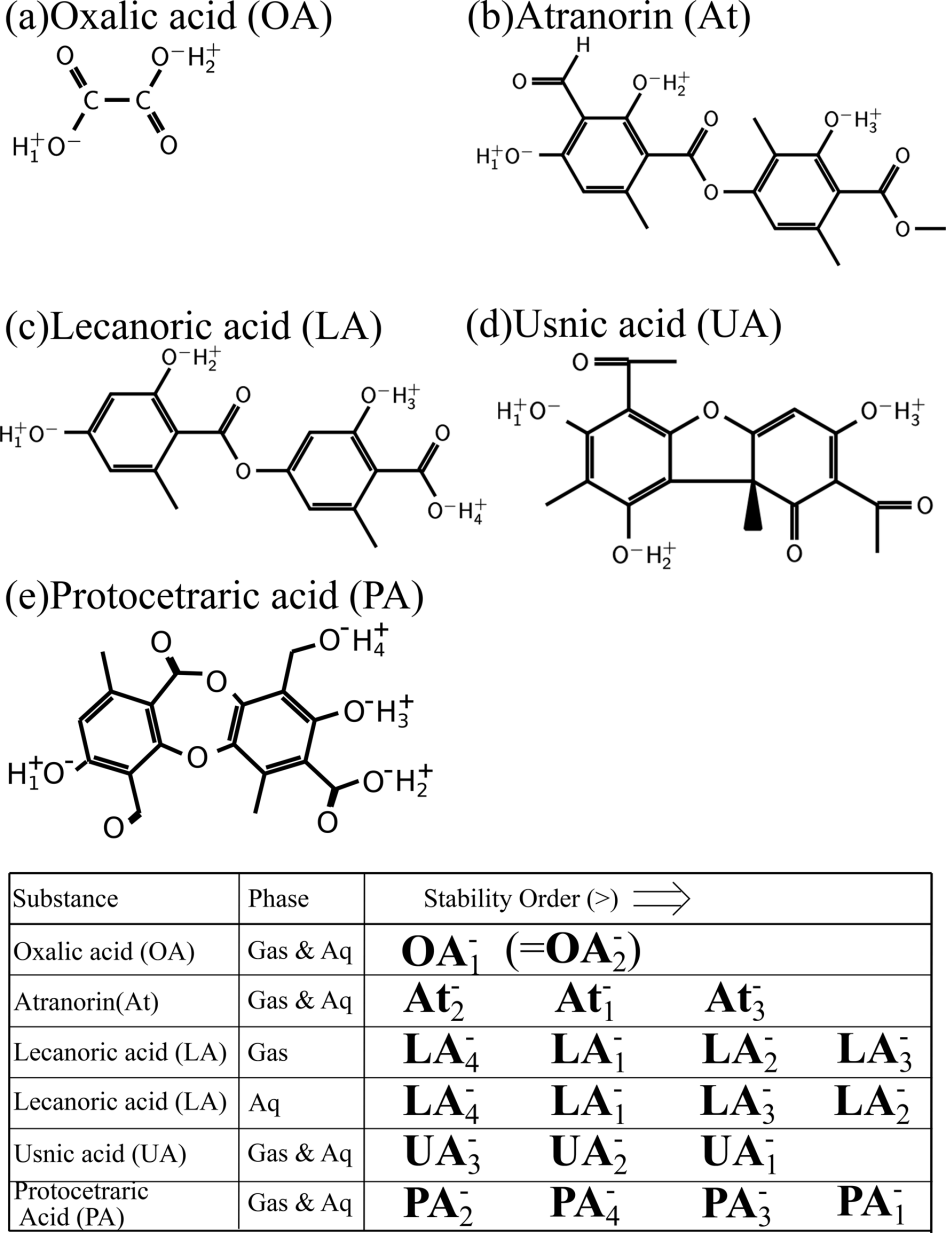
Lichen metabolites with marked hydrogen ions involved in the deprotonation: (**a**) oxalic acid, (**b**) atranorin, (**c**) lecanoric acid, (**d**) usnic acid, and (**e**) protocetraric acid. We also note the stability order among the deprotonated forms of the molecules. For example, the H$_2$-deprotonated atranorin (**At**$^-_2$) is more stable than the H$_1$-deprotonated one (**At**$^-_1$), the latter being more stable than H$_3$-deprotonated one (**At**$^-_3$) both in the gas phase and in aqueous solution. In oxalic acid, the mono-deprotonated form can be reduced to only a single one due to its symmetry (**OA**$^-_1=$**OA**$^-_2$).

All the lichen metabolites studied in this work are polyprotic acids (see Fig. 5), of which acidic character can be determined computationally. For example, in usnic acid, the problem was addressed also experimentally in Refs.^77–80^. In this work, in order to determine the acidic characters of all the target metabolites, we perform B3LYP/6-31G(d,p)/PCM calculations for every possible mono-deprotonated form of the lichen metabolites, and calculate their energies in the gas phase and the free energies in aqueous solution. While in oxalic acid the mono-deprotonated form can be reduced to only a single one due to its symmetry (**OA**$^-_1=$**OA**$^-_2$), we need to compare the stability order on all the distinguished deprotonation forms in the other metabolites. For atranorin, the H$_2^+$-deprotonated form (we note **At**$^-_2$, and see Fig. 5 for the numbering of protons) is more stable than the H$_1^+$-deprotonated one (**At**$^-_1$), and the latter form is more stable than H$_3^+$-deprotonated one (**At**$^-_3$) both in the gas phase and in aqueous solution, i.e., the stability order is **At**$^-_2>$**At**$^-_1>$**At**$^-_3$. For lecanoric acid (LA), we find **LA**$^-_4>$**LA**$^-_1>$**LA**$^-_2>$**LA**$^-_3$ in the gas phase and **LA**$^-_4>$**LA**$^-_1>$**LA**$^-_3>$**LA**$^-_2$ in aqueous solution, respectively. Similarly in usnic acid (UA), the order as **UA**$^-_3>$**UA**$^-_2>$**UA**$^-_1$ is found both in the gas phase and the aqueous solution, and in protocetraric acid (PA), the order as **PA**$^-_2>$**PA**$^-_4>$**PA**$^-_3>$**PA**$^-_1$ is found both in the two phases. In the following, we consider only the mono-deprotonated molecules, but we mention that oxalic acid and atranorin (by their experimental pK$_a$ values) are mainly present in di-deprononated forms in physiological pH range. Therefore, their results for the gas-phase complexation energies and the aqueous-solution complexation free energies are added in Figs. S3 and S4.

**Figure 6:**
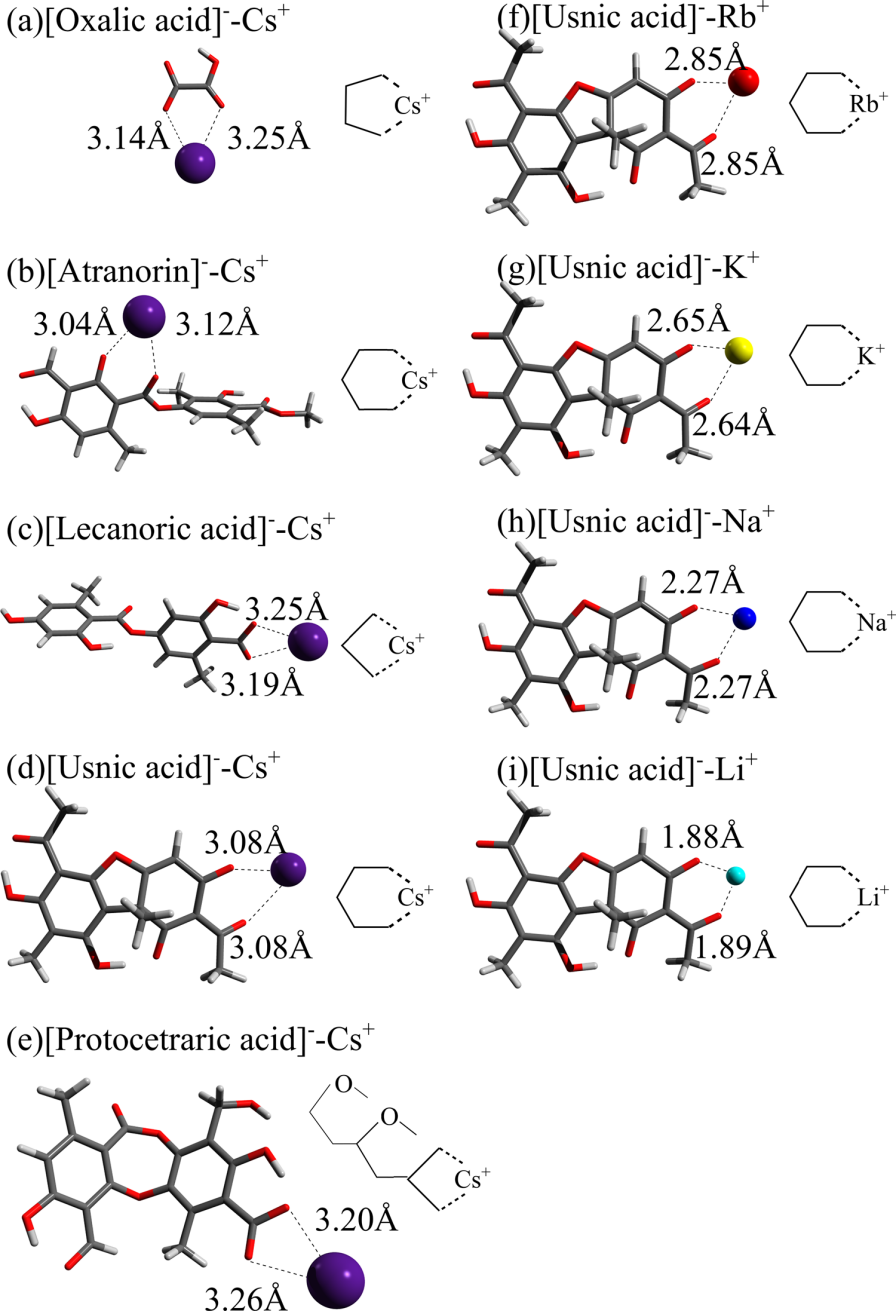
The most stable structures for the Cs-cation complexes of the deprotonated lichen metabolites: (**a**) oxalic acid, (**b**) atranorin, (**c**) lecanoric acid, (**d**) usnic acid, (**e**) protocetraric acid as well as those for the deprotonated usnic acid complexed with (**f**) Rb$^+$, (**g**) K$^+$, (**h**) Na$^+$, and (**i**) Li$^+$ in aqueous phase. The distances from the cesium cation to the nearest oxygen atom of the molecules are also indicated. The cation is seen to be complexed by forming a four-membered ring in (**c**) and (**e**) with two directionally aligned OH, a five-membered ring in (**a**), and a six-membered ring in (**b**), (**d**) and (**f**)–(**i**).

In the present study, we mainly perform structure search calculations for any complexation combinations of the mono-deprotonated lichen metabolites with the alkali-metal cations. The most stable structures for the Cs salts of oxalic acid, atranorin, lecanoric acid, usnic acid, and protocetraric acid in aqueous phase are shown in Fig. 6a–e. The distances between the Cs cation and the coordinated O atom(s) of the ligand are also depicted. The Cs$^+$-O distances are found to be between 3.0 and 3.3 Å. The structures for the Rb$^+$-Li$^+$ salts of usnic acid as the most stable forms in aqueous phase are also shown in Fig. 6f–i. Here, we notice that all the alkali-metal cations are found to be coordinated with two oxygen atoms. We also find that for the other deprotonated molecules, oxalic acid, atranorin, lecanoric acid, and protocetraric acid salts, not shown here, all the alkali-metal cations are similarly coordinated to two oxygen atoms.

**Figure 7:**
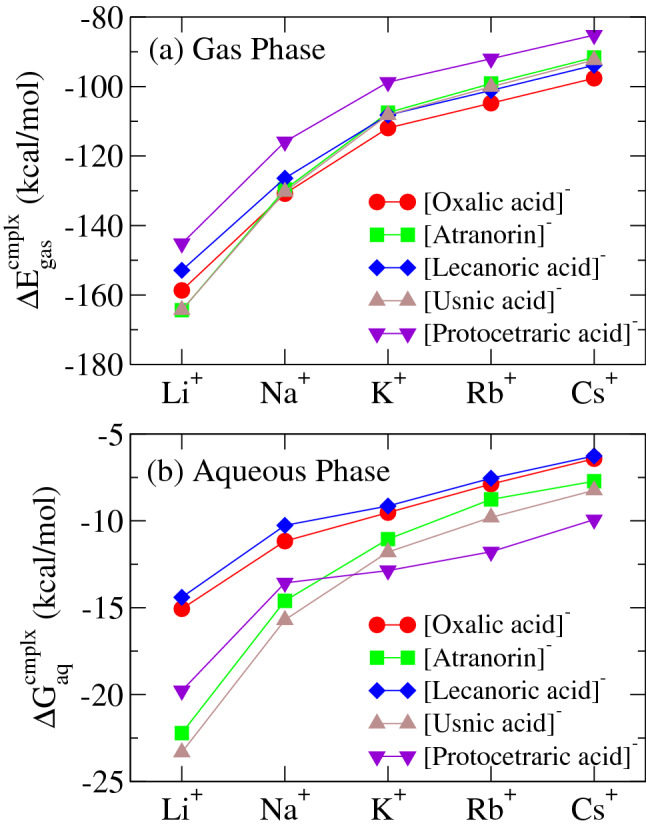
(**a**) Gas-phase complexation energies between the deprotonated oxalic acid, atranorin, lecanoric acid, usnic acid, and protocetraric acid molecules and the Cs, Rb, K, Na, and Li cations. (**b**) Aqueous solution complexation free energies between the deprotonated molecules and the alkali-metal cations.

The gas-phase complexation energies $\Delta E^{\mathrm {cmplx}}_{\mathrm {gas}}$ and the aqueous-solution complexation free energies $\Delta G^{\mathrm {cmplx}}_{\mathrm {aq}}$ between the deprotonated molecules and the alkali-metal cations are shown in Fig. 7a and b, respectively. In Fig. 7a, the gas-phase complexation energies are found to become more negative and larger for lighter alkali-metal cations, implying that these molecules favor complexations with lighter cations. We also find that all these molecules are found to complex the alkali-metal cations with almost similar strength, although protocetraric acid is found to complex them somewhat weakly. Such a trend can not be observed for the aqueous-solution complexation free energies in Fig. 7b, i.e., the deprotonated atranorin, usnic acid and protocetraric acid are found to complex the alkali-metal cations more strongly than the other molecules in aqueous phase, due to the subtle balance between the hydration free energies for the deprotonated molecules and their alkali-metal-cation complexes, as is illustrated in the supplemental information Sec.4 for the cases of the Cs$^+$ complexes. As schematically shown in Fig. 6, we find that the deprotonated atranorin and usnic acid are complexed with the alkali-cation by forming a six-membered X$^+$–O–C–C–C–O–X$^+$ ring (X$^+$ is an alkali-metal cation) in the most stable states, while two other molecules are done by forming a five-membered X$^+$–O–C–C–O–X$^+$ or four-membered X$^+$–O–C–O–X$^+$ ring, with one or two carbon atoms less compared with the six-membered ring. Hence, we suggest that the formation of such six-membered rings plays a key role in forming more stabilized complexes with alkali cations in aqueous solutions. We stress that the above difference becomes remarkable only in aqueous phases, in which distributed charge is significantly screened by the presence of water molecules, and then geometrical stabilization like the six-member ring just remains. This finding that usnic acid and atranorin form alkali-salt complexes in stronger manner is considered to be significant in physiological sense as discussed in “Discussion”. On the other hand, protocetraric acid forming a four-membered ring, is considered to complex the alkali-metal cation with peculiar strong binding due to the hydrogen-bond chain like formation of aligned multiple hydroxyl groups as shown in Fig. 6. This peculiar stability only in aqueous phase as seen in Fig. 7 is significantly specific for heavier ions than K, while its stability is weaker than atranorin and usnic acid for lighter Li and Na.

**Figure 8:**
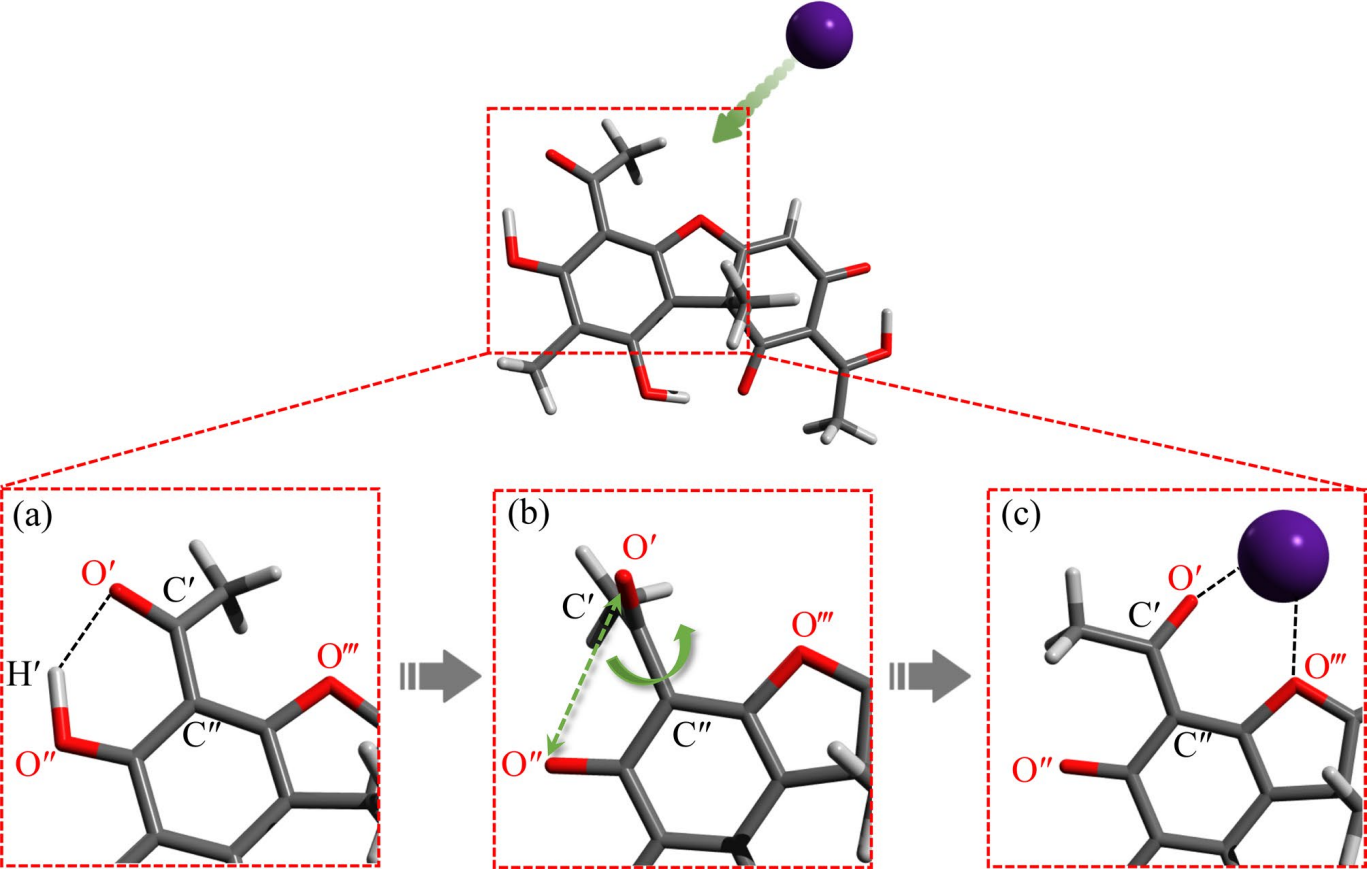
(**a**) In neutral situation, O′ is weakly hydrogen-bound with H′. (**b**) When the deprotonation of H′ occurs, the -COCH$_3$ possesses rotational freedom around the C′–C″ bond. (**c**) When the cation comes to complex with the molecule, an additional stabilization occurs due to the repulsive nature of the force between the O′ and O″ atoms, and the six-member ring emerges as the complex form.

In more details, we found that the deprotonated usnic acid shows slightly more stable than that of atranorin, i.e., that of usnic acid is the best absorber for light alkali-cations among four typical metabolites as shown in Fig. 7b. The above result stems from that usnic acid utilizes a conformational flexibility in forming salt complex being different from atranorin. We find atomistically that the conformational flexibility of the –OCCH$_3$ moiety plays an important role in this stabilization in the usnic acid salts. This moiety would possess rotational freedom around the C′–C″ bond as indicated in Fig. 8, depending on the surrounding cation configuration as follows. In the neutral situation, O′ is weakly hydrogen-bounded with the hydrogen H′ as shown in Fig. 8a. When the deprotonation occurs and subsequently an alkaline cation instead approaches, C′–C″ rotates as shown in Fig. 8b, and O′ forms the double-coordinated chelating complex with O‴ as shown in Figs. 8c and 6d. Thus, the alkali-metal cations are eventually coordinated to two oxygen atoms. Such a conformal flexibility is just intrinsic to the usnic acid in contrast to other molecules, and allows the usnic-acid-alkali salts to reach easily their own most stable structure without any activation energy. This brings about more strong complexation ability for usnic acid compared to other metabolites. To our knowledge, these findings are the first microscopic explanation on the strong complexation stability for alkali metal salts of usnic acid. Although there has been no direct experimental evidence consistent with these results, we stress that the usnic acid has been actually known to microscopically possess a high complexation ability with metal ions.

## Discussion

In this section, we discuss the calculated alkali-metal complexation energies in the gas phase and the free-energies in aqueous solution for five major metabolites, oxalic acid, usnic acid, atranorin, lecanoric acid, and protocetraric acid in their neutral and deprotonated states. In the neutral cases, all the molecules were found to energetically adsorb alkali-cations and form complex molecules in both gas and aqueous phases. This is clearly because all the molecules have electronegative oxygen atoms. This finding suggests that lichen intercellular spaces filled with their metabolites are able to easily catch alkali-cations in both gas and aqueous environments. More specifically, the calculations in the neutral cases revealed that protocetraric acid located in the medullary layer is the best adsorber among the five metabolites in both phases. In addition, lecanoric acid located in medullary layer shows higher stabilities than metabolites in other layers, although the energy differences are not so significant. This is marked contrast to the deprotonated cases, in which the complexation ability of lecanoric acid distinctively becomes lower than usnic acid and atranorin in aqueous phase. Those of usnic acid and atranorin exceed over that of protocetraric acid except for heavy cations. In deprotonated cases, usnic acid and atranorin are found to form six-member ring including adsorbed alkali cation chelated by two oxygen atoms, while four or five member ring occurs in cases of other metabolites. The six-member ring is energetically more stable, and then, the distances between oxygen and alkali-cations in the case are clearly shorter than those of four- and five-membered ones. These results indicate that usnic acid and atranorin are able to make more stable salts with alkali-cations in aqueous phases. Indeed, it is well-known that metal salts of usnic acid and atranorin are formed inside various lichen species^77–80^.

The above results enable to explain why usnic acid or atranorin abundantly exist in the upper cortex layer and lecanoric acid and protocetraric acid mainly locate at the lower medullary layer. We speculate that usnic acid and atranorin well serve as metal-cation absorbers in relatively high pH solution phases, in which significant amounts of metal-cations dissolve, while protocetraric acid and lecanoric acid work under low to neutral pH conditions, implying that it is effective for conditions in the medullary layer. Such a molecular based strategy on alkali-metal cation stress should be physiologically useful on lichens whose life is passive to hard environmental conditions. Furthermore, it is quite interesting for protocetraric acid located in medullary layer to have specifically strong catching ability on heavier alkali ions as seen in Fig. 7b. Such a chemical specificity should be advantageous for protecting the medullary layer homeostasis. Thus, we argue that the present type of calculations can explain inter-species differences in metal stress tolerance and heavy-metal retention ability. Indeed, there have been some reports on inter-species differences in Cs biomonitoring characters^6,81^. Especially, Dohi et al.^15^ and Ramzaev et al.^82^ revealed that *F. caperata* containing protocetraric acid exhibits a good tendency as Cs biomonitoring species.

From all the above results, we found intriguing variety in complexation ability of the metabolites with alkali metals including Cs, but we could find no specific Cs selectivity like a famous pigment molecule, norbadione A^49,83^ richly contained in mushrooms such as *Bay Boletus*. These results suggest that lichens do not selectively assemble Cs, but retain any alkali cations in the form of stable salts in the extracellular regions if having enough metabolites to catch various metal cations, though it should be noted that we did not cover all the metabolites. In addition, the present results indicate that the lichen metabolite complexes with alkali cations do not frequently exchange their cations once Cs complex formation occurs. We postulate that passive and poor nutrient condition uptake enables to keep such complexes intact for a long time. Strong complexation abilities found in the present metabolites should significantly contribute to such long-term stability. This is also consistent with the insight that lichens can relatively keep various anthropogenic elements^19,84^ released by air pollution events including nuclear accidents. However, it should be noted that there are other mechanisms^19,84^of metal cation retentions in lichens. Further experimental and computational studies will be required in the future.

## Summary and conclusion

In this work, we computationally examined alkali-metal complexation stabilities of the primary and secondary metabolites in some lichens observed to retain radioisotopes of Cs in Fukushima prefecture in order to understand their bio-retention mechanisms. The target metabolites are oxalic acid, atranorin, lecanoric acid, usnic acid, and protocetraric acid, which differently distribute inside the lichen’s stratified layers. We performed quantum chemical calculations for the alkali-metal-cation complexes of these metabolite molecules in their neutral and deprotonated cases. We searched the most and quasi stable structures both in the gas and aqueous phases and calculated the gas-phase complexation energies and the aqueous-solution free energies for these molecule—alkali-metal-cation complexes. The obtained gas-phase complexation energies and aqueous-solution complexation free energies revealed that all the molecules favor cation complexations with the standard order Li$^+>$Na$^+>$K$^+>$Rb$^+>$Cs$^+$ for all conditions. This indicates that there is no Cs selectivity, but that the considered metabolites strongly retained all the passively uptaken alkali cations. More specifically, in the neutral case, protocetraric acid and lecanoric acid located in the medullary layer form alkali-metal-cation complexes stronger than the other neutral molecules in aqueous phases, while, in the same phase, the deprotonated usnic acid and atranorin form complexes with alkali-metal cations distinctively stronger than the other deprotonated molecules except for protocetraric acid on heavier alkali cations. Then, we found that usnic acid possesses the strongest complexation ability due to the conformational flexibility of its twofold –OCCH$_3$ moieties and deprotonated protocetraric acid with proximate OH array exhibits higher stabilities only on heavier ions than potassium. Hence, we suggest that under high alkali metal stress, usnic acid and atranorin inside upper cortex significantly contribute to the lichen’s metal retention while lecanoric acid and protocetraric acid located in medullary layer plays outstanding roles as last adsorbers. These findings in chemical properties enable to provide a reason as to why these five metabolites show different distributions inside lichen stratified layers. Moreover, such metabolite-specific differences may explain different radionuclide retention abilities. We believe that the adopted atomistic approach sheds new lights on the retention mechanism of Cs and other alkali-metal ions relevant with lichen’s biomonitoring, but also for the comprehension of lichen physiology.

## Supplementary Information

Supplementary Information.
