# Supplementary material for: Quantum chemical calculation studies toward microscopic understanding of retention mechanism of Cs radioisotopes and other alkali metals in lichens
Source: Sci Rep. 2021 Apr 15;11:8228. doi: 10.1038/s41598-021-87617-w (PMC8050294; doi:10.1038/s41598-021-87617-w)
Supplement: Supplementary file 1 — Supplementary Information. [file 41598_2021_87617_MOESM1_ESM.pdf]

Supplementary information for quantum chemical calculation studies toward microscopic understanding of retention mechanism of Cs radioisotopes and other alkali metals in lichens

Hiroya Suno[1,*], Masahiko Machida[1], Terumi Dohi[2], and Yoshihito Ohmura[3]

[1]Center for Computational Science & e-Systems (CCSE), Japan Atomic Energy Agency, 178-4-4 Wakashiba, Kashiwa 277-0871, Japan
[2]Sector of Fukushima Research and Development, Japan Atomic Energy Agency, 6-6 Sakae-machi, Fukushima, Fukushima 960-8031, Japan
[3]Department of Botany, National Museum of Nature and Science, 4-1-1 Amakubo, Tsukuba, Ibaraki 305-0005, Japan
[*]suno.hiroya@jaea.go.jp

1 Tautomerism in usnic acid

Tautomerism in usnic acid can be automatically taken into account by using GRRM. Here, we demonstrated the mostly favorable tautomers obtained by DFT calculations with Gaussian through using GRRM with MOPAC. Figure S1 shows the calculated results on usnic acid in aqueous phase. These four different tautomers agree well with those found by Galasso in Ref. 1. They are found to be stable in the order $\mathbf{UA}_a > \mathbf{UA}_b > \mathbf{UA}_c > \mathbf{UA}_d$. These structures can be automatically selected with the order in the present scheme. The metal complex stabilities of these tautomers are automatically taken into consideration in the present calculation work.

2 pK$_a$ of the lichen metabolites

The neutral molecule dominates in pH<pK$_a$ (<pk$_{a1}$ in polyprotic acid) for target molecule, while the deprotonation occurs in pH$\gtrsim$pK$_{a1}$. pK$_a$'s of some typical metabolites are known in the literature[2–6]. In this work, we discuss complexation stabilities of the metabolites mainly in physiological pH range around pH$\sim$7.4$\pm$1.5 with literature information of pK$_a$'s of the present target molecules. In Fig. S2, we summarize these protonation properties of oxalic acid, atranorin, lecanoric acid, usnic acid, and protocetraric acid in the pH range. The pK$_a$'s presented in Fig. S2 are taken from Ref. 2 for oxalic acid, Ref. 4 for atranorin, Ref. 5 for lecanoric acid, and Ref. 3 for usnic acid. For protocetraric acid, the pK$_a$'s given here are referred from Ref. 6, in which those of hypoprotocetraric acid are instead employed owing its structural close similarity to protocetraric acid.

3 Complexation of di-deprotonated metabolites

As seen from their pK$_a$'s, oxalic acid and atranorin exist rather in their di-deprotonated states than in their mono-deprotonated states in the physiological pH range as seen in Fig. S2. Therefore, we examine here complexation stabilities of these two metabolites in the di-deprotonated states. Figures S3 shows the results on the most stable structures for the Cs-cation complexes of the di-deprotonated lichen metabolites in aqueous solution. For both of these two di-deprotonated metabolites, the cesium cation is seen to be complexed with two oxygen atoms. Figure S4 shows their gas-phase complexation energies and aqueous solution complexation free energies on each alkali cation. For both of the two di-deprotonated metabolites, we see the stability order $Li^+ > Na^+ > K^+ > Rb^+ > Cs^+$ both in the gas phase and in aqueous solution. While the di-deprotonated oxalic acid complexes alikali-metal cations more strongly than the di-deprotonated atranorin in the gas phase, the order is inverted in aqueous phase.

4 Hydration free energies for the deprotonated molecules and their Cs^+ complexes

In Fig.7, we see that the deprotonated protocetraric acid possesses stronger complexation ability than some of the other deprotonated metabolites in aqueous solution, whereas the trend is fully reversed in the gas phase with the protocetraric acid's complexation ability being the weakest. This trend reversion can be explained from the hydration free energies for these metabolites. Table S1 shows the hydration free energies for the deprotonated molecules and their Cs^+ complexes as well as

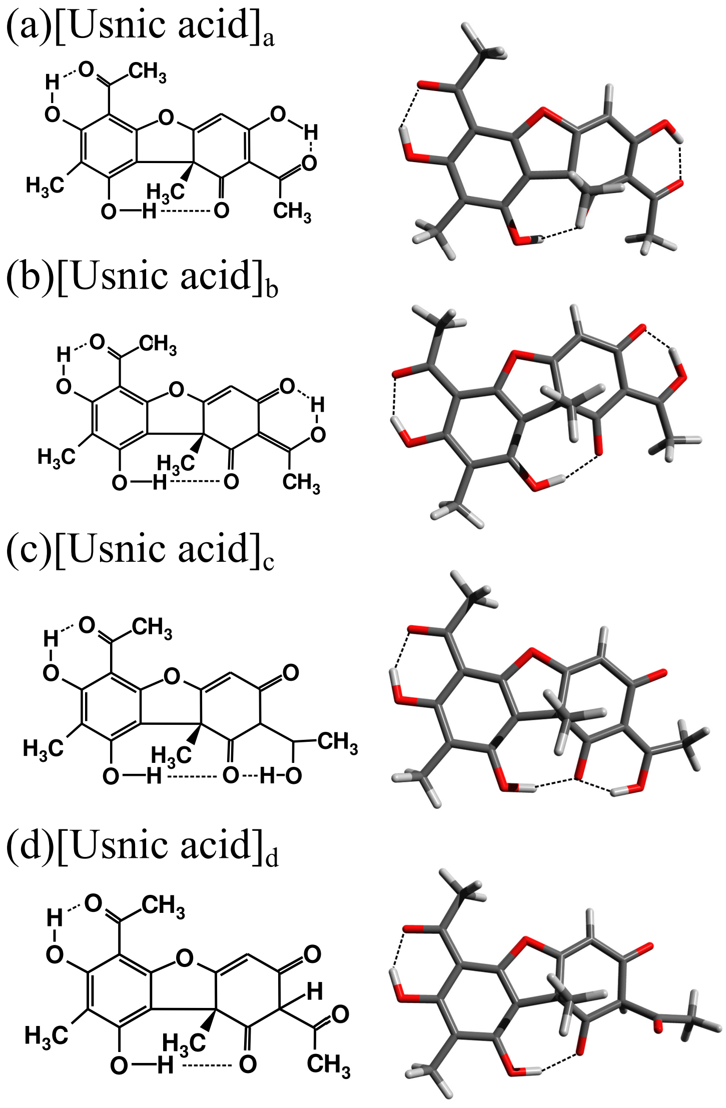

Figure S1. Molecular formulas and structures of the four most possible tautomers of usnic acid (UA) in aqueous solution. These tautomers are stable in the order $\mathbf{UA}_a > \mathbf{UA}_b > \mathbf{UA}_c > \mathbf{UA}_d$ in the present calculations. In figures on both the left and right sides, the dashed lines show hydrogen bonds.

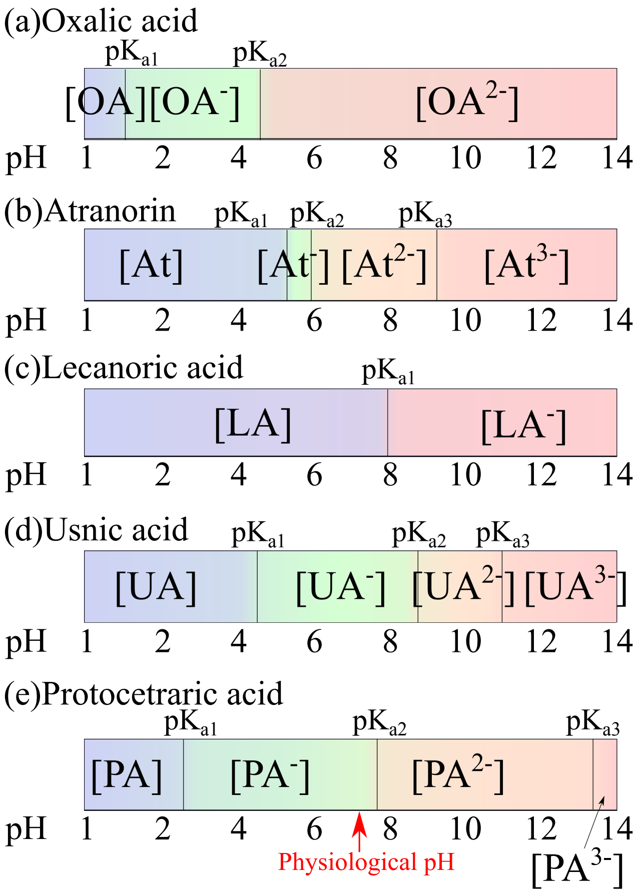

Figure S2. Protonation properties of (a) oxalic acid, (b) atranorin, (c) lecanoric acid, (d) usnic acid, and (e) protocetraric acid in the pH range. The physiological pH range is around pH$\sim$7.4$\pm$1.5.

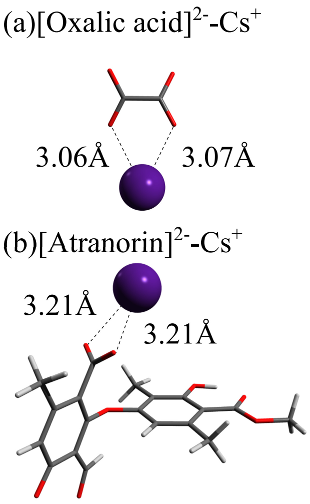

Figure S3. The most stable structures for the Cs-cation complexes of the di-deprotonated lichen metabolites in aqueous solution: (a) oxalic acid and (b) atranorin.

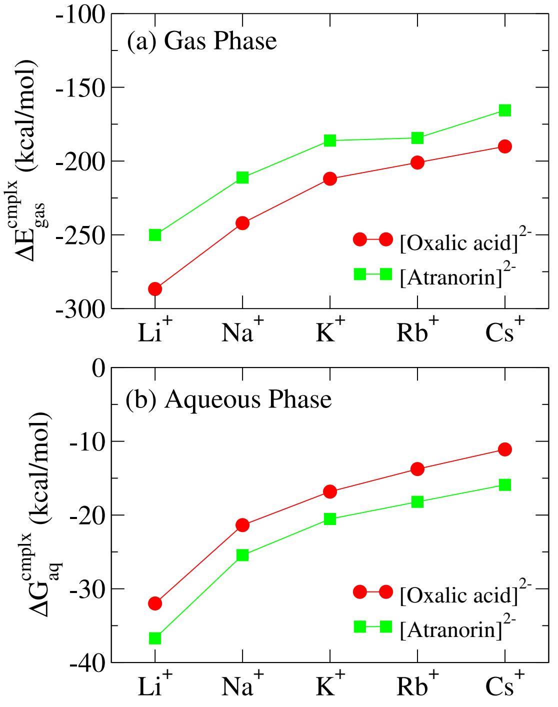

Figure S4. (a) Gas-phase complexation energies between the di-deprotonated oxalic acid and atranorin molecules and the Cs, Rb, K, Na, and Li cations. (b) Aqueous-solution complexation free energies between the different ligands and the different alkali-metal cations.

the contributions to the complexation free energies. For example, the contribution for the deprotonated oxalic acid is seen significantly small so that the aqueous-solution complexation free energy becomes small compared with the other molecules. Thus, oxalic acid is stronger than protocetraric acid in the gas phase, while protocetraric acid is stronger than oxalic acid in aqueous solution.

	[Oxalic acid]$^-$	[Atranorin]$^-$	[Lecanoric acid]$^-$	[Usnic acid]$^-$	[Protocetraric acid]$^-$
Molecule	−58.1	−50.1	−58.2	−59.3	−46.0
Cs^+ complex	−33.2	−32.5	−36.9	−40.4	−37.0
Contribution to ΔG_{aq}^{cmplx}	24.9	17.6	21.3	18.9	9.0

Table S1. Hydration free energies for the deprotonated molecules and their Cs^+ complexes, as well as the contributions to the complexation free energies, both in kcal/mol.

References

1. Galasso, V. Probing the molecular and electronic structure of the lichen metabolite usnic acid: A DFT study. *Chemical Physics* **374**, 138–145 (2010).

2. Clayton, G. D., Clayton, F. E. *et al. Patty's industrial hygiene and toxicology. Vol. 2A. Toxicology.* (John Wiley & Sons, Inc., Baffins Lane, Chichester, Sussex PO19 1DU, 1981).

3. Hauck, M., Jürgens, S.-R., Willenbruch, K., Huneck, S. & Leuschner, C. Dissociation and metal-binding characteristics of yellow lichen substances suggest a relationship with site preferences of lichens. *Annals of botany* **103**, 13—22 (2009). URL https://europepmc.org/articles/PMC2707280.

4. Ravaglia, L. M. *et al.* In vitro radical-scavenging activity, toxicity against A. salina, and nmr profiles of extracts of lichens collected from Brazil and Antarctica. *Química Nova* **37**, 1015–1021 (2014).

5. Bogo, D. *et al.* In vitro antitumour activity of orsellinates. *Zeitschrift für Naturforschung C* **65**, 43–48 (2010).

6. Bay, M. V. *et al.* Theoretical Study on the Antioxidant Activity of Natural Depsidones. *ACS omega* **5**, 7895–7902 (2020).
